# Fertilization, but Not Post-Implantation Development, Can Occur in the Absence of Sperm and Oocyte Beta1 Integrin in Mice

**DOI:** 10.3390/ijms232213812

**Published:** 2022-11-09

**Authors:** Nour El Houda Mimouni, Côme Ialy-Radio, Anne-Lyse Denizot, Isabelle Lagoutte, Michaela Frolikova, Katerina Komrskova, Sandrine Barbaux, Ahmed Ziyyat

**Affiliations:** 1 Université Paris Cité, CNRS, INSERM, Institut Cochin, F-75014 Paris, France; 2 Service d’Histologie, d’Embryologie, Biologie de la Reproduction, AP-HP, Hôpital Cochin, F-75014 Paris, France; 3 Laboratory of Reproductive Biology, Institute of Biotechnology of the Czech Academy of Sciences, BIOCEV, Prumyslova 595, 252 50 Vestec, Czech Republic; 4 Department of Zoology, Faculty of Science, Charles University, BIOCEV, Vinicna 7, 128 44 Prague, Czech Republic

**Keywords:** integrin beta1, *Stra8-Cre*, sperm, fertilization, ultrasound

## Abstract

Fertilization is a complex process that requires successive stages and culminates in the adhesion/fusion of gamete membranes. If the question of the involvement of oocyte integrins has been swept away by deletion experiments, that of the involvement of sperm integrins remains to be further characterized. In the present study, we addressed the question of the feasibility of sperm–oocyte adhesion/fusion and early implantation in the absence of sperm β1 integrin. Males and females with β1 integrin-depleted sperm and oocytes were mated, and fertilization outcome was monitored by a gestational ultrasound analysis. Results suggest that although the sperm β1 integrin participates in gamete adhesion/fusion, it is dispensable for fertilization in mice. However, sperm- and/or oocyte-originated integrin β1 is essential for post-implantation development. Redundancy phenomena could be at the origin of a compensatory expression or alternative dimerization pattern.

## 1. Introduction

A crucial step in fertilization is the sperm–oocyte contact that allows the two gametes to fuse and create a zygote. Many questions remain open about the molecules that bring the gametes’ membranes together. For several years, only a few proteins were considered essential for gamete adhesion/fusion: first, the CD9 tetraspanin [1,2,3], then the sperm IZUMO1 immunoglobulin [4] and the GPI-AP JUNO, which is the IZUMO1 oocyte receptor [5]. Over the past two years, six sperm factors essential for mammalian fertilization, SPACA6, TMEM95, SOF1, FIMP and DCST1/DCST2, have been identified using the CRISPR /Cas9 approach [6,7,8,9] (reviewed in [10]). However, this multiplication of factors does not seem to be sufficient to explain the gamete adhesion/fusion process, first because the mechanisms of action of these different actors are not fully known, particularly with regard to the most recently identified molecules, and because there are certainly other molecules to be discovered. Very recently, MAIA, also known as Fc receptor-like 3, has been demonstrated to supersede JUNO as IZUMO1 receptor during human fertilization [11]. These latest findings reinforce the idea of the existence of large and organized molecular complexes on each of the gamete plasma membranes, encouraging not only the identification of new players but also the reconsideration of some that have been sidelined. Integrins are included in the latter category during the fertilization process. Indeed, these were first considered essential; in particular α6β1 integrin has been described as a receptor for sperm ADAMs [12]. Conditional invalidation experiments with the *Itga6* gene [13] and then of the *Itgb1* gene [14] at the oocyte level showed that they were dispensable since the oocytes deleted from one or the other of these genes were fertilizable and female mice carrying one of these deletions were normally fertile.

In order to understand this apparent contradiction, we hypothesized that the presence of the β1 integrin subunit could be necessary on the membrane of one of the two gametes as occurs in myoblasts or other cells that fuse [15].

At first, we used synaptonemal complex protein 1 (*Sycp1)-Cre* and integrin subunit beta1 (*Itgb1) floxed* gene mice mating to generate sperm conditional knockout (KO) mouse, but this mouse did not show good excision efficiency at the point where males reproduced normally, with either wild-type (WT) or *Itgb1* KO oocyte (*Zp3-Cre ^+/^*^−^ *Itgb1 ^flox/flox^*) females, although in vitro, sperm showed a low fertilization rate compared with controls. *Zp3-Cre,* where the Cre is expressed under the control of the promoter of the *Zp3* gene encoding one of the three proteins of the zona pellucida (ZP) in mice, is highly effective as described [14], and accordingly, we never found integrin β1 protein on conditional KO oocytes. On the contrary, integrin β1 protein on the sperm of this *Sycp1-Cre* ^+/−^ *Itgb1 ^flox/flox^* model, that is a knockdown (KD), could still be detected with immunofluorescence and western blot [16]. We have identified two elements that could explain the presence of residual integrin β1 protein on KD sperm: first, the low efficiency of the Cre recombinase expressed under the control of the *Sycp1* promoter, especially when combined with the distance between the two Lox sites (~28 kb) in the first used *Itgb1 floxed* mice. Therefore, the question of whether the role of integrin β1 is essential or not in fertilization remains unanswered.

Here, we used a *Stra8-Cre* mouse [17] and a *Itgb1 floxed* gene mouse with a smaller distance (~0.6 kb) between the two LoxP sites [18]. Even though conditional KO (cKO) males continued to reproduce with a small decrease in the limit of significance when mated with WT females, there were no births when cKO males were mated with *Itgb1* KO females. On ultrasonography, we observed the presence of embryos and their peri-implantation lethality phenotype, which was previously described for total *Itgb1* gene KO [19,20]. Our findings indicate that integrin β1 is not essential for fertilization at the point of sperm–oocyte adhesion/fusion. However, there is no doubt about integrin β1′s participation during this process, as in its absence, in vitro fertilization rates were low, and sperm accumulated in the perivitelline space (PVS). In order to explain the present results, we propose that integrin β1could be resubstituted by another β integrin subunit, resulting in either another known integrin heterodimer or a novel dimerization. Nevertheless, these putative integrin heterodimers lacking β1 seem not functional during postimplantation embryo development, confirming that β1 integrin is essential during postimplantation.

## 2. Results

### 2.1. Efficiency of the Cre-Mediated Deletion of the Itgb1 Gene in Sperm and Oocytes

#### 2.1.1. Diagram of the Mating Used to Generate Conditional Knockouts on Sperm and Oocyte

**Figure 1:**
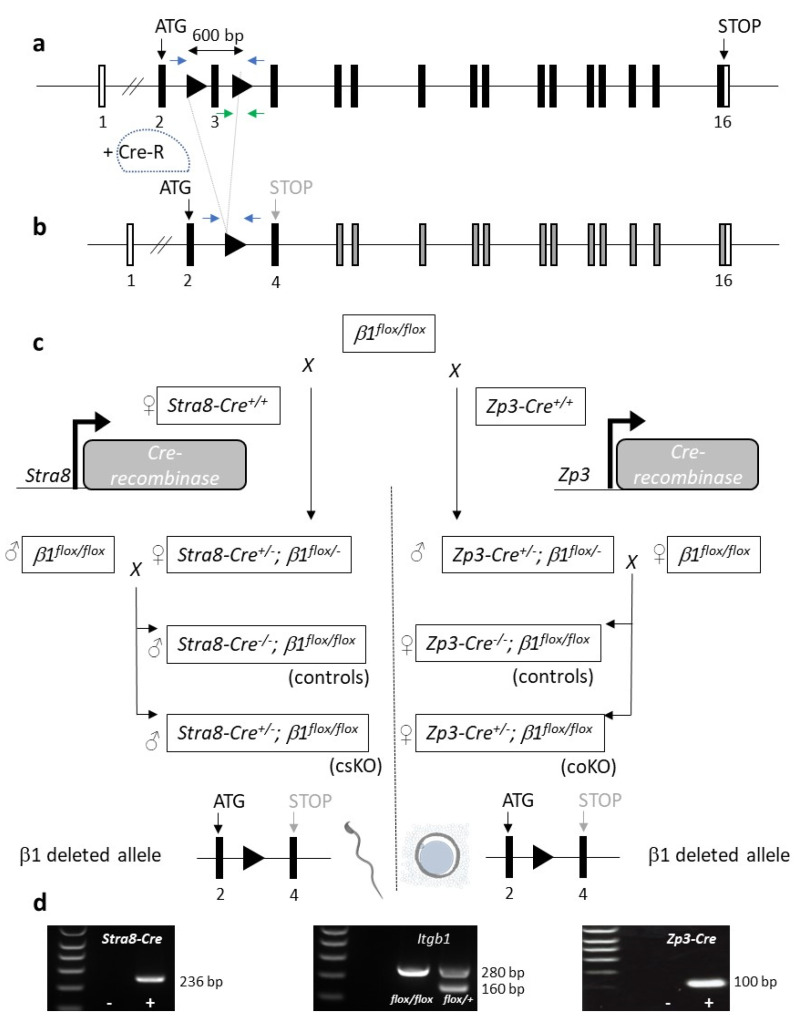
(**a**,**b**) Schematic representation of the *Itgb1 floxed* allele and excised allele respectively. (**c**) Mating using *Itgb1 ^flox/flox^* with *Stra8-Cre ^+/+^* females or *Zp3-Cre ^+/+^* males to obtain double heterozygous *Stra8-Cre ^+/^*^−^ *Itgb1 ^flox/-^* or *Zp3-Cre ^+/^*^−^ *Itgb1 ^flox/-^* that we mated with *Itgb1 ^flox/flox^* males or females respectively. Thanks to these crosses, the males of interest (*Stra8-Cre ^+/^*^−^ *Itgb1 ^flox/flo^*, csKO) and their controls (*Cre ^−/−^ Itgb1 ^flox/flox^*) (left) and the females of interest (*Zp3-Cre ^+/^*^−^ *Itgb1 ^flox/flox^*, coKO) and their controls (*Zp3-Cre ^−/−^ Itgb1 ^flox/flox^*) (right) were obtained. (**d**) The three gels show the presence of *Cre* whether it is under the control of the *Stra8* or *Zp3* promoters and the presence of the LoxP sites on one or two alleles of *Itgb1* gene.

Figure 1 illustrates a schematic representation of the *Itgb1 floxed* and deleted alleles (Figure 1a,b) [18] and the crossing scheme used in order to obtain the females (conditional oocyte knock-out or coKO) or the males (conditional sperm KO or csKO) in which the oocytes or the sperm are respectively invalidated for the *Itgb1* gene (Figure 1c). Three gels showing the presence of *Cre* gene under the control of the *Stra8* or *Zp3* promoters (Figure 1d left and right respectively) and the presence of the LoxP sites on one or two alleles of *Itgb1* gene (Figure 1d middle) are presented in Figure 1d. Floxed *Itgb1* gene mice were used as controls, and *Itgb1 floxed* mice expressing Cre represented the mice of interest to be tested.

#### 2.1.2. Efficiency of the Cre-Mediated Deletion of the Itgb1 Gene in Oocytes and Sperm

**Figure 2:**
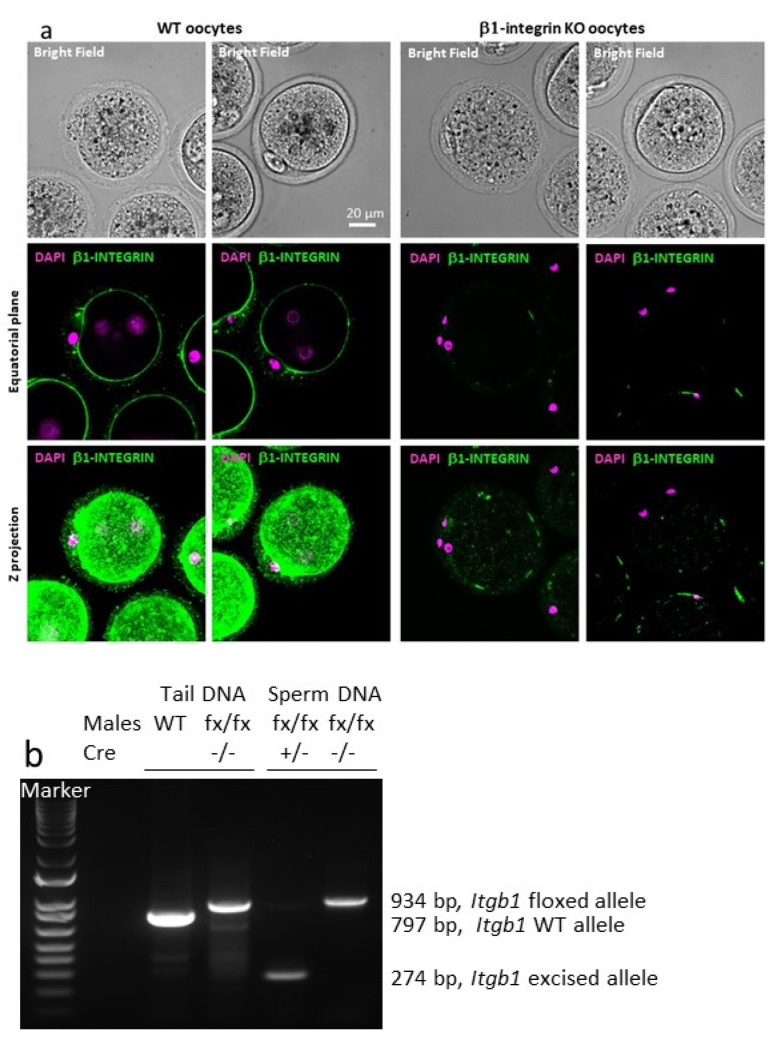
(**a**) Oocytes from *Zp3-Cre ^−/−^ Itgb1 ^flox/flox^* (WT oocytes) females express integrin beta1 at their surface while there is no staining on oocytes from *Zp3-Cre ^+/^*^−^ *Itgb1 ^flox/flox^* (KO oocytes). (**b**) *Itgb1* PCR on tail and sperm DNA. The same primers used gave 797 bp band on WT DNA, 934 bp in floxed (fx) tail DNA or *Cre*-negative sperm DNA and 274 bp on *Cre*-positive *Itgb1* excised allele sperm DNA.

As evaluated by immunofluorescence using a rat anti-mouse β1 integrin monoclonal antibody (MB1.2), all ovulated oocytes from conditional KO mice (*Zp3-Cre ^+/^*^−^ *Itgb1 ^flox/flox^*) that expressed Cre recombinase, contrary to control (CTRL, (*Zp3-Cre ^−/−^ Itgb1 ^flox/flox^*)) oocytes that did not express Cre recombinase, showed no staining as previously documented (Figure 2a).

Concerning the efficacy of the Cre recombinase on sperm, we looked at the status of the genomic DNA of spermatozoa in order to determine if the regions between the LoxP sites were indeed deleted in presence of Cre. When the Cre cohabited at the level of the male germ cells with the LoxP sites (two alleles floxed at the diploid stage), these were excised in the great majority since we could not detect the larger band corresponding to the persistence of the two LoxP sites and the sequence between them (Figure 2b).

### 2.2. In Vivo Evaluation of the Fertilizing Ability of Sperm from Stra8-Cre ^+/−^ Itgb1 ^flox/flox^ Males

**Figure 3:**
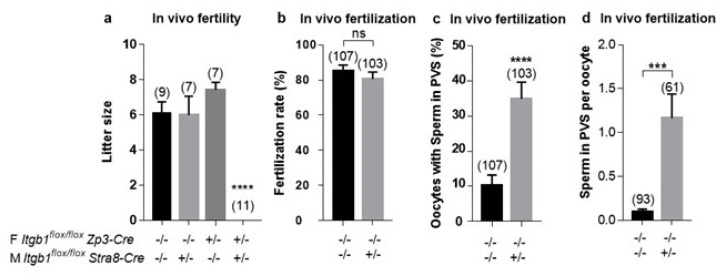
In vivo analysis of the fertilizing ability of *Stra8-Cre ^+/^*^−^ *Itgb1 ^flox/flox^* males (M) and *Zp3-Cre ^+/^*^−^ *Itgb1^flox/flox^* females (F). (**a**) Histogram representing the mean litter size of control (*Zp3-Cre ^−/−^ Itgb1 ^flox/flox^*) or conditional KO (*Zp3-Cre ^+/^*^−^ *Itgb1 ^flox/flox^*) females when mated with control (*Stra8-Cre ^−/−^ Itgb1 ^flox/flox^*) or conditional KO (*Stra8-Cre ^+/^*^−^ *Itgb1 ^flox/flox^*) males (numbers in parentheses represent the number of litters in each group). No statistical difference was revealed between the first three groups, whereas the coKO x csKO group gave no pups (*p* < 0.0001). (**b**) No significant difference in fertilization rate (mean ± s.e.m.) was obtained following in vivo fertilization (IVF) assay when control females were mated with control or csKO males. (**c**) Percentage of control oocytes (*Zp3-Cre ^−/−^ Itgb1 ^flox/flox^)* that accumulate sperm in their PVS after in vivo mating with control (*Stra8-Cre ^−/−^ Itgb1 ^flox/flox^*) or conditional KO (*Stra8-Cre* ^+*/*−^ *Itgb1 ^flox/flox^*) males (*p* < 0.0001). (**d**) Each oocyte inseminated by control sperm contained an average of 0.09 sperm; those inseminated with csKO sperm contained an average of 1.16 (*p* < 0.0003). In (**b**–**d**), the numbers in parentheses represent the number of used oocytes in each group. *** *p* < 0.001. **** *p* < 0.0001.

While no difference in litter size was observed during mating between the control males and the coKO or floxed females, mating using the csKO males gave normal-size litters with the control females but no live birth was obtained with the coKO females (Figure 3a).

In order to specify the step that explains this sterility, we mated csKO males with control or coKO females and recovered embryos the next day after checking the vaginal plugs. No difference in fertilization rate was noted, with 85.05 ± 3.46% and 80.58 ± 3.91%, respectively (Figure 3b). Nevertheless, we found the accumulation of spermatozoa in the PVS already described in our previous study [16]. While only 10% of oocytes inseminated with control sperm contained sperm in their PVS, over 34% of those inseminated with csKO sperm contained sperm in their PVS (*p* < 0.0001) (Figure 3c). In other words, each oocyte inseminated with control sperm contained an average of 0.09 sperm, whereas those inseminated with csKO sperm contained an average of 1.16 (12 times more, *p* < 0.0003) (Figure 3d).

### 2.3. Peri-Implantation Lethality of Embryos Resulting from Crosses of Stra8-Cre ^+/−^ Itgb1 ^flox/flox^ Males and Zp3-Cre ^+/−^ Itgb1 ^flox/flox^ Females

Using high-frequency ultrasonography, we followed the implantation and postimplantation development of embryos from control mating (Ctrl) and those from mating of Stra8-Cre ^+/−^ Itgb1 ^flox/flox^ males (csKO) and Zp3-Cre ^+/−^ Itgb1 ^flox/flox^ females (coKO). The control group showed normal implantation and development until birth with a 100% survival rate. In the group of KO mice, while implantation occurred normally, as attested by the presence of embryos at E6.5 (embryonic day), it was systematically followed by lethality at E10.5. Therefore, no births were obtained in this group (Table 1).

**Figure 4:**
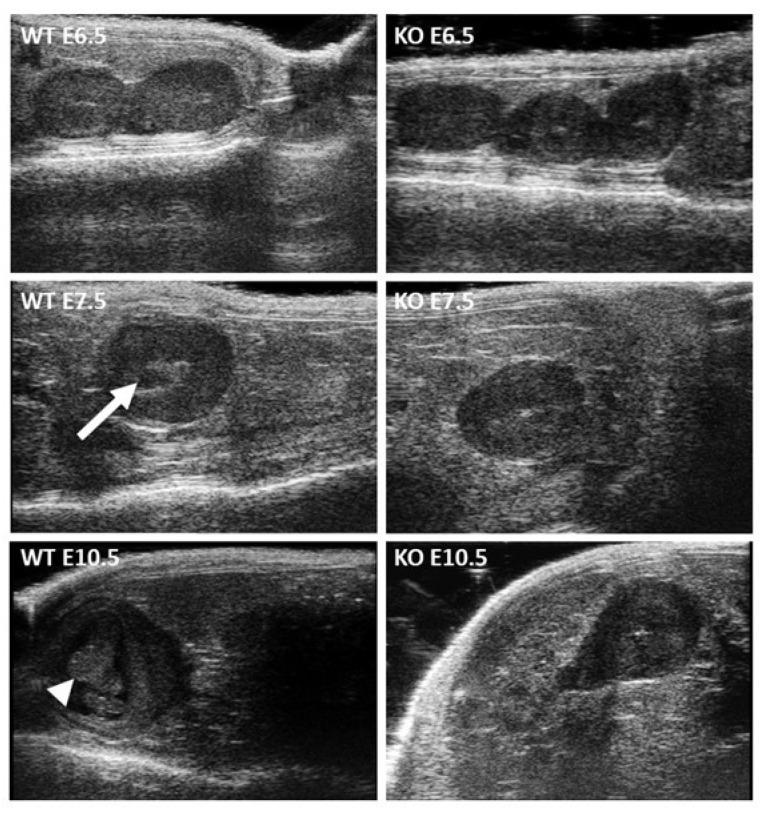
In vivo ultrasound microscopic observation of the embryonic development. During the gestation of control (WT, *Zp3-Cre ^−/−^ Itgb1 ^flox/flox^* females mated with *Stra8-Cre ^−/−^ Itgb1 ^flox/flox^* males) and KO (*Zp3-Cre ^+/^*^−^ *Itgb1 ^flox/flox^* females mated with *Stra8-Cre ^+/^*^−^ *Itgb1 ^flox/flox^* males) animals, the embryonic development was followed up by an in vivo ultrasonic method at E6.5, E7.5 and E10.5. No difference was observed at E6.5. At E7.5, less amniotic fluid was visible in the KO group compared to the control group (arrow). At E10.5, the embryo was no longer visible in the KO group compared to the control group (arrowhead). The viability of developing embryo was confirmed by the presence of heartbeats.

Figure 4 shows the in vivo ultrasound microscopic observation at E6.5, E7.5 and E10.5 in both groups. While there were no notable differences between the two groups with a homogeneous appearance of the embryo sac at E6.5, at E7.5 the amniotic fluid appeared as a dark spot in the middle of the amniotic sac (arrow) in control embryos but not in KO embryos. It was at E10.5 that the difference became obvious, with an embryo developing in a central position (arrowhead) surrounded by amniotic fluid (dark) in the case of the controls, while the amniotic sac was smaller and without any embryo in the case of the KO group. The viability of a developing embryo was dynamically confirmed by the presence of heartbeats in the control group and their absence in the KO group. In keeping with the fact that Itgb1 total deletion in mice results in inner cell mass failure and total peri-implantation lethality [19,20], the lethality of pups in our experiments demonstrated that Itgb1-deleted oocytes had to be fertilized by a sperm containing a deleted Itgb1 allele.

### 2.4. In Vitro Evaluation of the Fertilizing Ability of Stra8-Cre ^+/−^ Itgb1 ^flox/flox^ Sperm

**Figure 5:**
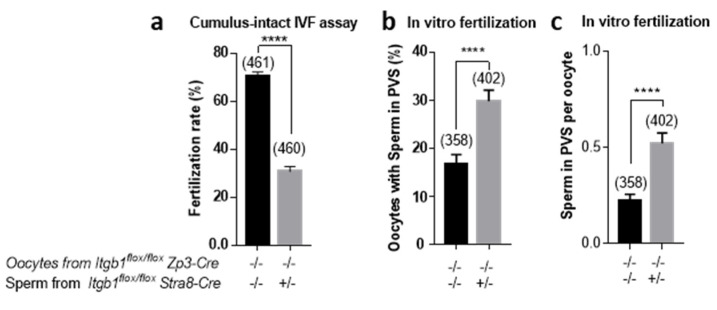
In vivo analysis of the fertilizing ability of sperm from *Stra8-Cre ^+/^*^−^ *Itgb1 ^flox/flox^* males when inseminated with oocytes from *Zp3-Cre ^−/−^ Itgb1 ^flox/flox^* females. (**a**) A significant difference in the fertilization rate (FR) (mean ± s.e.m.) was obtained following in vitro fertilization (IVF) in cumulus-intact assay between the control group (70%) and group using *Itgb1* gene-deleted sperm (30%) (*p* < 0.0001). (**b**) While 16% of the oocytes presented sperm in their PVS in the control group, 29% contained PVS sperm in the group of oocytes inseminated with sperm from *Stra8-Cre ^+/^*^−^ *Itgb1 ^flox/flox^* males (*p* < 0.0001). (**c**) The mean number of sperm per oocyte was 0.22 ± 0.03 in the control group versus 0.52 ± 0.05 in the group using deleted sperm (*p* < 0.0001). The numbers in parentheses represent the number of used oocytes in each group. **** *p* < 0.0001.

For in vitro fertilization, sperm from *Stra8-Cre ^−/−^ Itgb1 ^flox/flox^* males and oocytes from *Zp3-Cre ^−/−^ Itgb1 ^flox/flox^* females were used in the control group. In this group, the fertilization rate was 70.07 ± 2.13% (n = 461) and dropped to 30.65 ± 2.15% (n = 460) when sperm from *Stra8-Cre ^+/^*^−^ *Itgb1 ^flox/flox^* were used (*p* < 0.0001), indicating the participation of sperm integrin β1 in the fertilization process (Figure 5a). In this latter case, 29.85 ± 2.28 % of oocytes (n = 402) showed additional sperm in their PVS compared with the 16.76 ± 1.97 % oocytes (n = 358) in the control group (*p* < 0.0001), confirming that the deleted sperm had more difficulty binding to and fusing with oocytes than the controls (Figure 5b). The mean number of PVS sperm per oocyte was 0.22 ± 0.03 in the control group versus 0.52 ± 0.05 in the group using deleted sperm (*p* < 0.0001) (Figure 5c).

## 3. Discussion

To study the function of integrin β1 during in vivo fertilization, we intended to generate *Itgb1* sperm conditional KO mice. Due to the partial efficiency of the Cre activity under the control of the promoter of the male germ cells specific gene, *Sycp1*, we obtained a *Itgb1* Knock-down mouse line in the first place. Therefore, we could not clearly determine the role of integrin β1 in fertilization. It was suggested that the inefficiency of the Cre was due to the distance between the two LoxP sites (~28 kb).Therefore, we used a *Stra8-Cre* mouse [17] and another *Itgb1 floxed* gene mouse with a smaller distance (~0.6 kb) between LoxP sites [18]. This developed mouse line was also used in crossing with the *Zp3-Cre* mouse line to obtain oocyte-conditional KO mice. The effectiveness of Cre under *Zp3* promoter control was already shown by us and others [14,16], and we further confirmed it by immunofluorescence. Indeed, oocytes from more than 10 conditional KO mice were tested, and 100% of the oocytes showed no integrin β1 signal, unlike the controls. For the males, we performed PCR on sperm DNA. Indeed, the male germ line is the only one to have both the floxed β1 alleles and the Cre expressed under the control of the *Stra8* promoter. PCR showed only the presence of the excised alleles indicating a very high efficiency of the Cre recombinase.

At this point, this *Stra8-Cre ^+/^*^−^ *Itgb1 ^flox/flox^* male model appeared to be the right tool for revealing the function of sperm integrin β1 during in vivo fertilization. The fact that *Itgb1*-conditional KO males continued to reproduce normally when they were mated with WT females indicated either that sperm integrin β1 was not necessary for in vivo fertilization or that oocyte integrin β1 could compensate for sperm-originated integrin β1 absence. In order to test the oocyte compensation hypothesis, we mated sperm-conditional KO males with *Itgb1*-oocyte KO females. Indeed, we did not obtain any births. Here again, two explanations were possible: the observed sterility could have been due to either a defect in fertilization or a defect in development. Using the same mating scheme, we retrieved oocytes from females that exhibited a vaginal plug the day after mating. The fertilization rates were the same in the KO group as in the controls, thus demonstrating that the absence of the integrin β1 subunit in both sperm and oocytes was not an obstacle to fertilization. The only anomaly that we noted was the presence of spermatozoa in the PVS, with a higher frequency than in the controls. This phenotype indicated that sperm lacking integrin β1 could take longer to fuse with the oocyte. The possible delay in triggering the cortical reaction, which normally follows fusion and prevents polyspermy [21], could explain the entry of additional sperm into PVS. These results are in favor of a theory of participation of β1 integrin in fertilization in terms of optimization. In support of this hypothesis, in vitro fertilization, where conditions are not optimal as in vivo, showed a significant decrease in fertilization rates when control oocytes were inseminated by cKO sperm, in addition to a greater accumulation of cKO spermatozoa in PVS.

The hypothesis that remained to be tested in order to explain sterility was that of embryonic lethality. With ultrasonography, we observed the presence of embryos and their peri-implantation lethality phenotype, which was previously described for total *Itgb1* gene KO [19,20]. These findings indicated two points. First, the Cre under the control of the *Stra8* promoter was more efficient than that under the control of the *Sycp1* promoter that we had used before [16]. Among the thirty pups generated for ultrasonography experiments, all resorbed; that is, none had inherited an intact (still floxed) *Itgb1* gene. Second, integrin β1 participates as well, but it is not essential for fertilization to take place, at least not in vivo. A hypothesis that could explain this apparent contradiction is related to a possible redundancy either by the de novo expression of another beta integrin subunit or by a new dimerization in the absence of the β1 integrin subunit. This hypothesis could also explain why the in vitro fertilization rate was lower when Cre was less effective using the *Sycp1-Cre* mouse line [16]. We propose that when Cre is strongly efficient (*Stra8-Cre*), there is low or no integrin β1 mRNA and protein expression, setting up up a functional compensatory integrin heterodimer system in the absence of integrin β1 subunit. Further investigations are needed to verify this redundancy hypothesis. For example, comparative studies of the transcriptome and/or the proteome of gametes (oocytes and spermatozoa) lacking beta1 and control gametes that express integrin β1 could make it possible to detect genes or proteins expressed only in the context of the absence of integrin β1 subunit.

## 4. Materials and Methods

### 4.1. Ethics Statement

All animal experiments were performed in accordance with national guidelines for the care and use of laboratory animals. Authorizations were obtained from local (C2EA-34, Comité d’éthique en matière d’expérimentation animale Paris Descartes) and governmental ethical review committees via APAFiS Application (Autorisation de projet utilisant des animaux à des fins scientifiques), Authorization APAFIS #14124-2017072510448522 v26, A. Ziyyat (2018–2023).

### 4.2. Generation of Oocyte and Sperm Itgb1 Conditional Knockout Mice

The floxed *Itgb1* gene mice [18] (JAX stock #004605) were mated with the transgenic mice expressing the Cre recombinase under the control of the *Zp3* promoter [14,22] (JAX stock #003651) or the *Stra8* promoter [17] (JAX stock #017490). Heterozygous mice for *floxed Itgb1* and *Zp3-Cre* or *Stra8-Cre* were mated with *floxed Itgb1* mice to generate mice homozygous for the floxed *Itgb1* gene and expressing Cre. Female mice with both the *Zp3-Cre* transgene and homozygous floxed *Itgb1* gene (*Zp3-Cre ^+/^*^−^ *Itgb1 ^flox/flox^*) were used for in vivo mating. Male mice with the *Stra8-Cre* transgene and homozygous floxed *Itgb1* gene (*Stra8-Cre ^+/^*^−^ *Itgb1 ^flox/flox^*) were used for in vivo mating and IVF assays. For all experiments, controls used were *Stra8*- or *Zp3*- *Cre ^−/−^ Itgb1 ^flox/flox^* males or females respectively.

Genotyping of mice was performed by PCR amplification (GoTaq^®^ DNA Polymerase, Promega, Madison, WI, USA) on DNA extracted from tail biopsies (NucleoSpin^®^ Tissue, Macherey-Nagel, Düren, Germany) using the following primers: 5′- CGGCTCAAAGCAGAGTGTCAGTC -3′ and 5′- CCACAACTTTCCCAGTTAGCTCTC-3′ for floxed *Itgb1* gene detection and one or the other of these two pairs of primers: 5′- GCGGTCTGGCAGTAAAAACTATC -3′ and 5′- GTGAAACAGCATTGCTGTCACTT-3′ or 5′- AGATGCCAGGACATCAGGAACCTG-3′ and 5‘-ATCAGCCACACCAGACACAGAGATC-3′ for *Cre* gene detection under the control of *Zp3* or *Stra8* promoters. The first primer pair gives an amplification of 160 bp for WT *Itgb1* and 280 bp for floxed *Itgb1* (Figure 1a, green arrows). The second and the third ones give amplimers of 100 bp or 236 bp respectively.

### 4.3. Immunofluorescence of Mouse Embryos

Zona-intact early embryos from control or conditional KO mice were fixed during 20 min in 4% formaldehyde at room temperature (RT) and washed three times in PBS-1% BSA. For detection of the integrin β1, embryos were incubated with the primary antibody (MB1.2 at 20 µg/mL; Chemicon International, Temecula, CA, USA) for 1 h at RT followed by Alexa Fluor^®^ 488 goat anti-rat IgG secondary antibody incubation (10 µg/mL, Molecular Probes, Invitrogen, Illkirch, France). Embryos were then incubated with 5µg/mL Hoechst 33342 (Invitrogen H3570) in PBS-1% BSA for 30 min and washed in PBS-1% BSA and mounted in PBS in Nunc™ Lab-Tek™ Chamber Slide System (Thermo Fisher Scientific, Illkirch, France). Control immunofluorescent studies were performed using isotype (IgG2a) (Serotec-Bio-Rad, Marnes-la-Coquette, France) as primary antibody or Alexa Fluor^®^ 488 goat anti-rat IgG alone. Observations and acquisitions were carried out on an inverted spinning disk microscope using oil-immersion objectives X40. Final images were processed, Z-projected upon request, and merged using ImageJ.

### 4.4. PCR on Mouse Tail and Sperm DNA

DNA was extracted from sperm pellets and mouse tail biopsies (NucleoSpin^®^ Tissue, Macherey-Nagel, Düren, Germany). The amplification was performed thanks to primers located upstream of the LoxP5′ in intron 2 (5′-TATAACCCGCAGAACAATAGG-3′) and downstream of LoxP3′ in intron 3 (5′-CCACAACTTTCCCAGTTAGCTCTC-3′) and GoTaq^®^ DNA Polymerase (Figure 1a, blue arrows). Amplimers of 797, 934 or 274 bp were obtained for the WT, floxed or excised allele respectively.

### 4.5. In Vivo Mating, Gamete Preparation and in vitro Fertilization

Four groups of mating were used: KO males (i.e., males expressing Cre under the control of the *Stra8* promoter at the heterozygous state) with KO females (i.e., females expressing Cre under the control of the *Zp3* promoter at the heterozygous state), KO males with control females, control males with KO females and control males with control females. All mice were homozygous for the *Itgb1* floxed gene. Mice were aged from 8 to 14 weeks and were housed as one male and one female per cage. In each group, litter size was assessed and compared to other groups.

WT and conditional KO female mice (5–8 week-old) were superovulated with 5 IU pregnant mare serum gonadotropin (PMSG) and 5 IU human chorionic gonadotropin (hCG, Intervet, Beaucouze, France) 48 h apart. Fourteen hours after hCG injection, animals were sacrificed by cervical dislocation. Cumuli oophori were collected by tearing the ampulla wall of the oviduct, placed in Ferticult medium (FertiPro, Belgium) supplemented with 3% bovine serum albumin (BSA) and maintained at 37 °C under 5% CO_2_ in air under mineral oil (Sigma, St. Louis, MO, USA). For zona-free in vitro fertilization (IVF) assay, oocytes were freed from the cumulus cells by 3–5 min incubation at 37 °C with hyaluronidase (Sigma) in M2 medium (Sigma). Oocytes were rinsed and kept in Ferticult medium at 37 °C under 5% CO_2_ atmosphere under mineral oil. Zona pellucida (ZP) was then dissolved with acidic Tyrode’s (AT) solution (pH 2.5, Sigma) under visual monitoring. The zona-free eggs were rapidly washed in medium and kept at 37 °C under 5% CO_2_ atmosphere for 2 to 3 h to recover their fertilizability.

Mouse spermatozoa were obtained from the cauda epididymis of control or KO conditional male mice (8 to 14 weeks old) and capacitated at 37 °C under 5% CO_2_ for 90 min in a 500 µL drop of Ferticult medium supplemented with 30 mg/mL BSA, under mineral oil.

Cumulus-intact or zona-free oocytes were inseminated for 3 h in a 50 µL drop of medium with capacitated spermatozoa at a final concentration of 1 × 10^6^/^mL^ or 1 × 10^5^/^mL^ respectively. Then, they were washed and directly mounted in Vectashield/DAPI for observation under UV light (Zeiss Axioskop 20 microscope). They were considered fertilized when the oocytes showed a fluorescent decondensed sperm head within their cytoplasm.

### 4.6. High Frequency Ultrasonography Implantation and Survival Assessment

Gestations were obtained by crossing oocyte *Itgb1*-conditional KO females (*Zp3-Cre ^+/^*^−^ *Itgb1 ^flox/flox^*) with sperm *Itgb1*-conditional KO males (*Stra8-Cre ^+/−^ Itgb1 ^flox/flox^*). Females were used to collect phenotypic data from the gestation. Females from the control group (*Zp3*- *Cre ^−/−^ Itgb1 ^flox/flox^*) were crossed with control males (*Stra8*-*Cre ^−/−^ Itgb1 ^flox/flox^*). Implantation and survival rates were obtained using high-frequency (40 MHz) ultrasonography (VEVO 2100 with MS-550D probe, Visulasonics, Toronto, Canada). Briefly, a chemical hair remover was used to eliminate abdominal hair. Ultrasonographic contact gel was used to ensure contact between the skin surface and the transducer. Body temperature, electrocardiographic and respiratory profiles were monitored using ultrasound device’s integrated heating pad and monitoring device (THM150, Indus Instruments, Webster, TX, USA). The implantation and the survival rates were determined early in the gestation, at E6.5, E7.5 and E10.5. At these stages, the small size of embryos permits a fluent count and resorbed embryos are also visible. During each examination, the number of implanted embryos in each uterine horn as well as their status (alive or dead) were assessed.

### 4.7. Statistical Analysis

Results are expressed as mean ± sem of at least three independent experiments. For statistical analysis, one-way ANOVA multiple-comparisons test or t-test were performed using GraphPad Prism version 7.00 for Windows (GraphPad Software, La Jolla California, CA, USA). Differences were considered statistically significant when *p* < 0.05.

## Figures and Tables

**Table 1:** Survival rate after crossbreeding of csKO males and coKO females or control (Ctrl) mice measured by ultrasound microscopy.

Mating	Implanted Embryos at E6.5	Implanted Embryos at E10.5	Birth	Survival Rate
♀Ctrl × ♂♂Ctrl	5	5	5	100%
	5	5	5	
	5	5	5	
	8	8	8	
♀coKO × ♂♂csKO	9	0	0	0%
	7	0	0	
	5	0	0	
	7	0	0	

## Data Availability

Not applicable.
